# Cardiac Involvement in Facioscapulohumeral Muscular Dystrophy (FSHD)

**DOI:** 10.3389/fneur.2021.668180

**Published:** 2021-05-24

**Authors:** Allison Ducharme-Smith, Stefan Nicolau, C. Anwar A. Chahal, Kirstie Ducharme-Smith, Shujah Rehman, Keerthi Jaliparthy, Nadeem Khan, Christopher G. Scott, Erik K. St Louis, Teerin Liewluck, Virend K. Somers, Grace Lin, Peter A. Brady, Margherita Milone

**Affiliations:** ^1^Department of Cardiovascular Diseases, Mayo Clinic, Rochester, MN, United States; ^2^Department of Neurology, Mayo Clinic, Rochester, MN, United States; ^3^Mayo Clinic Graduate School of Biomedical Sciences, Rochester, MN, United States; ^4^The Royal Papworth Hospital National Health Service Trust, Cambridge, United Kingdom; ^5^Johns Hopkins Bloomberg School of Public Health, Baltimore, MD, United States; ^6^Department of Internal Medicine, Mayo Clinic, Rochester, MN, United States; ^7^Department of Biomedical Statistics and Informatics, Mayo Clinic, Rochester, MN, United States; ^8^Illinois Masonic Medical Center, Advocate Aurora Health, Chicago, IL, United States

**Keywords:** arrhythima, facioscapulohumeral dystrophy, FSHD, mitral valve prolapse, conduction

## Abstract

**Background:** Facioscapulohumeral muscular dystrophy (FSHD) is one of the most common muscular dystrophies and predominantly affects facial and shoulder girdle muscles. Previous case reports and cohort studies identified minor cardiac abnormalities in FSHD patients, but their nature and frequency remain incompletely characterized.

**Methods:** We reviewed cardiac, neurological and genetic findings of 104 patients with genetically confirmed FSHD.

**Results:** The most common conduction abnormality was complete (7%) or incomplete (5%) right bundle branch block (RBBB). Bifascicular block, left anterior fascicular block, complete atrioventricular block, and 2:1 atrioventricular block each occurred in 1% of patients. Atrial fibrillation or flutter were seen in 5% of patients. Eight percent of patients had heart failure with reduced ejection fraction and 25% had valvular disease. The latter included aortic stenosis in 6% (severe in 4% and moderate in 2%) and moderate aortic regurgitation in 8%. Mitral valve prolapse (MVP) was present in 9% of patients without significant mitral regurgitation. There were no significant associations between structural or conduction abnormalities and age, degree of muscle weakness, or size of the 4q deletion.

**Conclusions:** Both structural and conduction abnormalities can occur in FSHD. The most common abnormalities are benign (RBBB and MVP), but more significant cardiac involvement was also observed. The presence of cardiac abnormalities cannot be predicted from the severity of the neurological phenotype, nor from the genotype.

## Introduction

Facioscapulohumeral muscular dystrophy (FSHD) is one of the most common muscular dystrophies and predominantly involves facial and shoulder girdle muscles (1), with a degree of phenotypic variability among affected individuals. There are two genetically distinct but clinically indistinguishable subtypes of FSHD, termed FSHD1 and FSHD2. The majority of patients have FSHD1, which results from a contraction of the *D4Z4* microsatellite repeat containing the *DUX4* pseudogene on the long arm of chromosome 4. Development of FSHD additionally requires a permissive 4qA haplotype distal to the *D4Z4* microsatellite repeat (2). FSHD1 is inherited in an autosomal dominant fashion, with up to 30% of cases occurring *de novo*. The *D4Z4* contraction leads to translational de-repression of *DUX4* (3). This is generally thought to result from hypomethylation of the *D4Z4* repeats, though some studies have questioned this view (4, 5). There is also evidence that additional genetic mechanisms play a role in the development of FSHD (6, 7). Approximately 5% of FSHD patients have FSHD2, which is characterized by hypomethylation of the *D4Z4* locus in combination with a permissive 4qA haplotype, but without a *D4Z4* contraction (8–10). Many but not all FSHD2 patients harbor mutations in *SMCHD1* or, less frequently, in *DNMT3B* or *LRIF1* (11). Mutations in *SMCHD1* have also recently been associated with non-neuromuscular phenotypes (1, 12–14).

Occurrence of certain extramuscular manifestations, such as retinal vasculopathy and sensorineural hearing loss, are well-established in FSHD (15). Cardiac involvement however, which is common in many other muscular dystrophies (16), has not been fully characterized in FSHD. Cardiac screening and surveillance have not been recommended in FSHD patients without cardiac symptoms (15, 17, 18). Several case reports and cohort studies of genetically proven FSHD patients have identified cardiac abnormalities in FSHD patients. The most commonly identified cardiac abnormality has been incomplete right branch block (RBBB), detected in ~23–33% of patients (17, 19). Supraventricular tachyarrhyhmias have also been reported in approximatively 10% of FSHD patients (20, 21). A smaller number of patients exhibits ventricular arrhythmias, cardiac conduction system abnormalities such as interventricular conduction delay and atrioventricular (AV) block, focal myocardial fibrosis or fatty infiltration with preserved ejection fraction, or a hypertrophic cardiomyopathy phenotype (20–25). Limitations of the various published studies include absence of genetic confirmation in some cases, inconsistent correlation of cardiac abnormalities with the severity of the muscle disease or underlying genetic defect, association of arrhythmias with comorbidities and cardiac risk factors (26), and sometimes the small number of patients included.

Herein, we report on the frequency and nature of cardiac abnormalities observed in a large cohort of genetically proven FSHD patients evaluated at a single academic medical center. We investigated the frequency of cardiac symptoms, cardiac conduction abnormalities, and structural heart abnormalities in these patients and correlated cardiac abnormalities with the severity of muscle weakness and genetic findings.

## Methods

**Figure 1:**
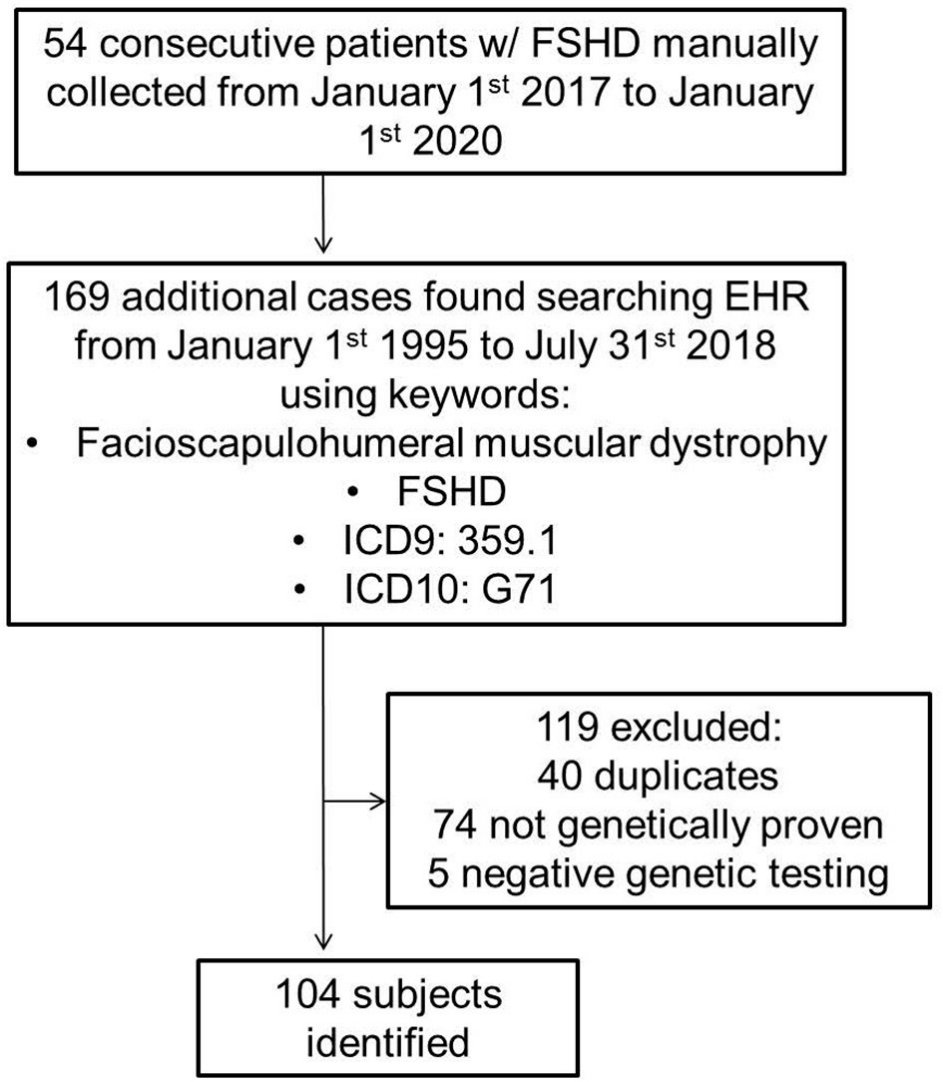
FSHD, facioscapulohumeral muscular dystrophy; EHR, electronic health record.

Using both a manually maintained registry and a search of electronic medical records, we identified all cases of FSHD evaluated at Mayo Clinic from January 1st 1995 to July 31st 2018 (Figure 1). The search was performed using a combination of keywords, and ICD9 and ICD10 codes (facioscapulohumeral muscular dystrophy, FSHD, 359.1, and G71). The inclusion criterion was a genetically proven diagnosis of FSHD1 or FSHD2. We excluded patients with a clinical diagnosis of suspected FSHD without genetic confirmation. A case note abstraction form with a pre-defined data dictionary was designed and piloted in REDCap (Research Electronic Data Capture; University of Vanderbilt, Tennessee, USA). REDCap is a secure, web-based application designed to support data capture for research studies, providing: (1) an intuitive interface for validated data entry; (2) audit trails for tracking data manipulation and export procedures; (3) automated export procedures for seamless data downloads to common statistical packages; and (4) procedures for importing data from external sources (27).

Data were manually abstracted and collected, including demographics, genetic testing results, age of onset of muscle weakness, degree of muscle weakness, age at diagnosis, cardiac symptoms and findings, and known independent cardiac risk factors (hypertension, hyperlipidemia, current or prior smoking, obesity, and diabetes mellitus). Muscle power was evaluated by manual muscle testing using the Medical Research Council (MRC) scale. A sum score was calculated using the facial muscles, weakest upper limb muscle and weakest lower limb muscle on each side (range 0–30). The following cardiac findings were collected and analyzed: echocardiograms, stress tests, and Holter monitoring data. In addition, electrocardiogram (ECG) data were electronically abstracted using MUSE (GE, Milwaukee, WI, USA). Mitral valve prolapse was defined as mitral valve displacement on echocardiogram of more than 2 mm above the mitral annulus in long-axis view (28).

Categorical variables are reported as frequencies and continuous variables with medians and interquartile ranges (IQR) (29). Continuous variables were compared using *t*-tests or non-parametric tests for non-Gaussian distributions. *T*-tests were performed between variables of interest (age, degree of muscle weakness, and size of the 4q deletion) and the cardiac outcome variables (conduction, structural abnormalities and cardiac risk). Categorical variables were compared using *X*^2^ or Fisher's exact test. A *p* < 0.05 was considered statistically significant for all analyses. Analyses were performed using SAS version 9.4 (SAS Institute, Cary, NC, USA) and R 3.4.2 (R Foundation for Statistical Computing, Vienna, Austria).

## Results

We identified 104 consecutive patients with genetically proven FSHD: 99 with FSHD1 and five with FSHD2. Table 1 summarizes demographics, symptoms, clinical and genetic findings, as well as cardiovascular risk factors of our cohort. The median age of onset was 26 years and 59% of patients were male. Seventy percent of patients were older than 18-years at symptom onset. The median age at molecular diagnosis was 45 years (IQR 29, 60). The median manual muscle testing score was 24 (IQR 24, 26). Seventy-one percent of patients had a typical distribution of weakness, involving the face and shoulder girdle muscles. Thirteen percent had shoulder girdle weakness with facial sparing. The remainder had atypical presentations, most commonly facial and predominant lower limb weakness. Among patients with FSHD1, the median EcoRI restriction fragment length was 29.5 kb (IQR 24, 33). Presence of the 4qA haplotype was confirmed in 42 patients. The remainder of patients underwent testing at a time when haplotyping was not commercially available or underwent testing in a laboratory that did not perform haplotyping. FSHD2 patients carried one of the following mutations in *SMCHD1*: c.724G>A (p.A242T, two patients), c.1273G>A (p.G425R), c.1787G>A (p.W596^*^), or c.1435C>T (p.R479^*^).

**Table 1:** Clinical data, genetic findings, and cardiovascular risk factors in facioscapulohumeral muscular dystrophy (FSHD) type 1 and 2 patients.

**Clinical and laboratory data**		**Population (*n =* 104)**
Age, years, median (IQR)		46 (29, 60)
Male, *n* (%)		61 (59%)
Onset of weakness, median age (IQR)		26 (15, 50)
Age at diagnosis, years, median (IQR)		45 (29, 60)
Disease duration at diagnosis, years, median (IQR)		14 (6, 23)
Manual muscle testing score, median (IQR)		24 (24, 26)
Pattern of weakness (*n*, %)	Typical	74 (71%)
	Facial-sparing	13 (13%)
	Atypical	15 (14%)
EcoRI restriction fragment length, Kb (IQR) (FSHD1 patients, *n =* 99)^*^		29.5 (23, 33)
**Cardiovascular risk factors**		
Hypertension, *n* (%)		36 (35%)
Hyperlipidemia, *n* (%)		28 (27%)
Current or prior smoking, *n* (%)		25 (24%)
Obesity, *n* (%)		29 (28%)
Diabetes mellitus, *n* (%)		10 (10%)
**Cardiac symptoms**		
Chest pain, *n* (%)		7 (7%)
Angina, *n* (%)		1 (1%)
Dyspnea, *n* (%)		24 (23%)
Palpitations, *n* (%)		11 (11%)
Syncope/presyncope, *n* (%)		0 (0%)

*IQR, interquartile range; Kb, kilobase range; FSHD, facioscapulohumeral muscular dystrophy*.

* *Five patients had FSHD2*.

Table 2 summarizes the cardiac findings. ECGs were performed in 70% (*n* = 73) of patients, 12% (*n* = 12) of whom had also had Holter evaluation. ECGs were obtained for cardiac surveillance in the setting of FSHD or for cardiac symptoms. ECGs were abnormal in 49% of the 73 patients in whom they were obtained. The most common abnormalities were RBBB and atrial fibrillation/flutter, which were detected in 7% (*n* = 5) of patients and 5% (*n* = 4) of patients, respectively. The patients with 2:1 AV block (*n* = 1) and first degree AV block (*n* = 5) were asymptomatic. The single patient with complete AV block had a cardiac device, which was a dual chamber pacemaker for complete heart block occurring post-surgical aortic valve replacement. No patient had an implantable cardioverter defibrillator or biventricular pacemaker. Amongst those with Holter monitoring, premature ventricular complexes (PVC) were recorded in 50% (*n* = 6), non-sustained VT in 50% (*n* = 6), premature atrial complexes in 25% (*n* = 3), and sustained VT in 8% (*n* = 1). Among the patients with PVCs, one patient had a 6% burden of ventricular events, while the burden was <1% in the other five patients. Among the seven patients with non-sustained or sustained VT, five had an echocardiogram, which showed: left atrial enlargement and apical hypokinesis in one patient, severe right ventricular enlargement as well as severe aortic stenosis (trileaflet valve) and tricuspid regurgitation in one patient who had also RBBB, prior aortic valve replacement and subsequently normal ejection fraction and valvular function in one patient, and no structural abnormalities in the remaining two patients. Four patients had atrial fibrillation on either ECG or Holter.

**Table 2:** Cardiovascular findings in facioscapulohumeral muscular dystrophy (FSHD) type 1 and 2 patients.

	**Evaluated (*n*)**	**FSHD**	**Normal values**
**Electrocardiogram**	73		
PR duration, ms, median (IQR)		154 (140, 170)	100–210
QRS duration, ms, median (IQR)		92 (87, 100)	80–100
1st degree AV block^†^, *n* (%)		5 (7%)	
2:1 AV block, *n* (%)		1 (1%)	
Complete AV block, *n* (%)		1 (1%)	
Atrial fibrillation/flutter, *n* (%)		4 (5%)	
Left and right atrial enlargement, *n* (%)		8 (11%)	
Left and right axis QRS deviation, *n* (%)		2 (3%)	
Left ventricular hypertrophy, *n* (%)		4 (5%)	
Incomplete right bundle branch block, *n* (%)		4 (5%)	
Right bundle branch block, *n* (%)		5 (7%)	
Left bundle branch block, *n* (%)		1 (1%)	
**Holter monitor**	12		
Premature atrial complexes, *n* (%)		3 (25%)	
Premature ventricular complexes, *n* (%)		6 (50%)	
Non-sustained VT, *n* (%)		6 (50%)	
Sustained VT, *n* (%)		1 (8%)	
Atrial fibrillation, *n* (%)^*^		1 (8%)	
**Echocardiogram**	53		
Left ventricular posterior wall width, cm, median (IQR)		1.0 (0.8, 1.0)	0.6–1.1
Left ventricular internal diameter diastole, cm, median (IQR)		4.6 (4.4, 5)	4.2–5.8
Pulmonary pressure, mmHg (IQR)		26 (23.5, 30)	8–20
Aortic regurgitation, *n* (%)		2 (4%)	
Aortic stenosis, *n* (%)		3 (6%)	
Mitral valve prolapse, *n* (%)		5 (9%)	
Bicuspid aortic valve, *n* (%)		1 (2%)	
Tricuspid regurgitation, *n* (%)		3 (6%)	

*AV, atrioventricular; IQR, interquartile range; VT, ventricular tachycardia*.

* *This patient also had atrial fibrillation on electrocardiogram*.

† *1st degree AV block was defined as a PR interval > 210 ms*.

Echocardiography was obtained in 51% (*n* = 53) of patients (of these, all 53 had ECGs and 12 also had Holter evaluation). Echocardiography was abnormal in 42% (*n* = 22) of these, as summarized in Table 2. Eight percent (*n* = 4) of these patients had heart failure with reduced ejection fraction; three of these four patients had ejection fractions of 30–40% while one had an ejection fraction of 50%. Two of the four patients had known mild bystander coronary artery disease (CAD), which was disproportionate to the degree of left ventricular dysfunction. One of these patients with heart failure had a complex medical history and the heart failure was attributed to her ischemic dilated cardiomyopathy and pulmonary hypertension. Two had sepsis-related cardiomyopathy, both of which normalized with resolution of sepsis. Valvular abnormalities were present in 24% (*n* = 13) and some of these consisted of more than one valvular lesion. Nine percent (*n* = 5) had mitral valve prolapse and 6% (*n* = 3) had mitral regurgitation, all of which were mild. Six percent had aortic stenosis (*n* = 3), two of which were severe requiring aortic valve replacement, while the third was moderate. Four percent had aortic regurgitation (*n* = 2) which was moderate in severity. Finally, 6% had tricuspid regurgitation (*n* = 3), which was mild in severity with the exception of one patient. In this case, the patient had both severe aortic stenosis and severe tricuspid regurgitation. The latter patient is the same patient described above with heart failure, reduced ejection fraction and several other structural abnormalities in the setting of scleroderma, dilated cardiomyopathy and pulmonary hypertension, as well as genetically confirmed FSHD 1.

Forty-four percent of the cohort (*n* = 46) had no cardiac risk factors (which included patients who had no identified obesity, hypertension, hyperlipidemia, smoking, or diabetes). In this cohort without underlying cardiac risk factors, 13% had a conduction abnormality (*n* = 6) which included intermittent right bundle branch block (*n* = 2), frequent premature atrial contractions (*n* = 1), left axis deviation (*n* = 2). Sixteen percent (*n* = 7) had a structural abnormality, including MVP (*n* = 2), atrial enlargement (*n* = 3), and mitral regurgitation (*n* = 2).

Eight patients had early onset FSHD, defined as either facial weakness before age five or shoulder girdle weakness before age 10 (30). Six of these eight patients had both ECG and echocardiogram. One had left atrial enlargement, one had right atrial enlargement, two had biatrial enlargement, and one had right ventricular hypertrophy with right axis deviation. In addition, one of these patients had a small secundum ASD. None of these abnormalities appeared clinically relevant, as the patients were all asymptomatic and none had atrial fibrillation or flutter detected during our follow-up period.

There was no significant difference in the frequency of structural and conduction abnormalities between classical and facial-sparing or other atypical presentations. Of the five patients with FSHD2, only two had an ECG and echocardiogram. Both patients had intermittent RBBB and normal echocardiograms.

Only 11% of patients (*n* = 11) underwent stress testing, which was requested for reported dyspnea on exertion. Exercise stress test data revealed ischemia in two patients, aged 74 and 79 years. The first was felt to have stable and non-progressive symptoms managed well with medical therapy. The second underwent coronary artery catheterization, which demonstrated diffuse coronary artery disease requiring coronary artery bypass grafting.

There were statistically significant associations between age and presence of any cardiac risk factor (*p* < 0.001), presence of cardiac structural abnormalities (*p* = 0.01), but not presence of cardiac conduction abnormalities (*p* = 0.4). Individuals with cardiac risk factors and structural abnormalities tended to be older.

There were no significant associations between cardiac structural abnormalities and degree of muscle weakness (*p* = 0.5), or size of the 4q35 deletion in FSHD1 (*p* = 0.4). Similarly, there were no associations between cardiac conduction abnormalities and degree of muscle weakness (*p* = 0.6), or size of the 4q35 deletion in FSHD1 (*p* = 0.6). There were also no associations between cardiac abnormalities and degree of muscle weakness or size of the 4q35 deletion when males and females were analyzed separately, among the patients without cardiac risk factors, or among patients with early onset (*p* > 0.3 for all comparisons).

In our cohort, 4% (*n* = 4) of patients experienced a stroke, either ischemic (*n* = 3) or hemorrhagic (*n* = 1). Three of these patients had structural heart abnormalities, while one had a normal echocardiogram. As most of our patients presented to our clinic only for establishing the diagnosis of the underlying neuromuscular disease, only 43 patients were seen in follow-up one or more years after the diagnosis. Seven of these 43 patients (16%) followed in the cohort died at a median age of 68 years (IQR 56, 79). The causes of death for these seven patients were pneumonia, disseminated histoplasmosis, stroke, hypercapnic respiratory failure from muscular dystrophy, pancreatic cancer, cardiac arrest secondary to severe gastrointestinal bleed, and unknown cause (*n* = 1 each).

## Discussion

We describe the cardiac findings in a large cohort of genetically proven FSHD patients assessed at a single center. In these patients, we identified cardiac conduction and structural abnormalities, as well as a higher than expected prevalence of mitral valve prolapse. These cardiac abnormalities did not correlate with patient age or degree of muscle weakness, and, similarly to previous studies, the cardiac abnormalities did not correlate with the size of the 4q deletion in FSHD1 (17, 20, 21). We observed only an association between age and presence of cardiac risk factors, as expected in the general population. Two of 5 FSHD2 patients had incomplete RBBB but these patients were too few to assess prevalence of cardiac abnormalities in this subgroup.

Of the 73 patients (70%) who underwent an ECG, we found 7% had a complete RBBB, 5% had incomplete RBBB, and 3% had higher degree AV block. This is higher than expected in the general population, where the prevalence of incomplete and complete RBBB is 3% and 0.8%, respectively (31). Other studies have reported a much higher prevalence of incomplete RBBB (19-33%) in FSHD1 (17, 19, 20), but a slightly lower frequency of complete RBBB (4%) (17). However, none of these conduction abnormalities were clinically relevant. Meanwhile, the proportion of left bundle branch block in our cohort was similar to the general population (32). The mechanism of conduction abnormalities in FSHD is unknown. One could speculate that this may be related to abnormal vascular smooth muscle differentiation that selectively involves the His-Purkinje fibers (33). Alternatively this could be due to myocardial tissue changes, including focal fibrosis and fat infiltration described in FSHD1 via cardiovascular magnetic resonance (24), which may serve as a substrate for arrhythmias (22).

In the absence of traditional cardiac risk factors, we found a high prevalence of conduction abnormalities (13%). This is similar to Trevisan et al., who found that 12% of their cohort of 83 patients had cardiac involvement, which mostly consisted of arrhythmic disturbances (21). It is unknown whether the cardiac abnormalities detected in our patients were static or progressive, as most patients were not followed longitudinally. Limited literature is available in this regard. An 8-year follow-up study in 27 FSHD1 patients, who were mainly found to have incomplete and complete RBBB, showed no progression of the ECG abnormalities in the majority of patients (17). Longitudinal follow-up would be of interest, as the autonomic modifications reported with disease progression, such as increased sympathetic activity and decreased parasympathetic activity, could increase the risk of cardiac arrhythmias over time (34).

We also observed that four of the 73 (5%) patients who underwent ECG evaluation and six of 12 (50%) patients who underwent Holter had atrial fibrillation and non-sustained ventricular tachycardia, respectively. The non-sustained ventricular tachycardia was asymptomatic and shorter than 15 beats in all six patients, in the context of structurally normal hearts, and thus no further management was required. The prevalence of atrial fibrillation by age, in our patients, is consistent with that observed in the general population (35). Interestingly, we did not detect any supraventricular tachycardias in our cohort, which is in contrast to the literature where one of the main arrhythmogenic abnormalities observed in patients with FSHD was atrial tachycardia (21, 36). This may be due to the low frequency of Holter monitoring obtained in our cohort.

Interestingly, we found the frequency of MVP in our cohort was 9%, in comparison to 1–2% in the general population (37). This result may be skewed by the lack of echocardiographic data in all patients of this cohort. However, even assuming the remaining patients did not have MVP, its prevalence would still be higher than expected in the normal population. While MVP has been associated with Duchenne muscular dystrophy (38), to our knowledge, this is the first time an increased prevalence of MVP has been reported in FSHD. Mitral valve coaptation is complex, requiring appropriate valve leaflet length, and subvalvular apparati of chordae and papillary muscles. The mechanisms leading to prolapse include abnormalities of leaflet length, strength and shape, as well as annular disjunction. Subtle changes to cardiac muscle without overt dilatation or hypertrophy can result in papillary muscle dysfunction, which can contribute to MVP. One could speculate that minor dysfunction of the papillary muscles may have resulted in MVP. None of our patients had cardiac MRI to explore the possibility of focal fibrosis or fatty infiltration that could have led to MVP. On the other hand, the prevalence of bicuspid aortic valve (2%), aortic stenosis (6%), and tricuspid regurgitation (6%) was similar to that seen in the general population (39, 40). We observed no isolated cardiomyopathy, and the three FSHD patients with compromised ejection fraction had coexisting independent cardiovascular disease.

It is notable that four of eight patients with early-onset FSHD had atrial enlargement or ventricular hypertrophy, which had not been reported in other cohorts. Previous studies had identified conduction abnormalities in early-onset FSHD, but it remains uncertain whether these abnormalities occur at a higher frequency in early-onset FSHD compared to those with a more classical age of onset (20, 23, 41, 42).

The strength of our study is a cohort of genetically confirmed FSHD patients with comprehensively characterized neurological phenotypes. The limitations include those inherent to a retrospective design. Cardiac investigations were not obtained universally across the cohort to determine the extent of cardiac abnormalities in asymptomatic patients. As a result, the frequency of abnormalities in patients who underwent testing may be higher than in the broader FSHD population. Our study may also be underpowered to detect rare cardiac abnormalities. Follow-up data was not available for most patients, as they were evaluated for establishing the neuromuscular diagnosis, and subsequently received care locally.

In summary, we found no association between cardiac findings and genotype or severity of skeletal myopathy in FSHD. Baseline ECG abnormalities were benign and did not require cardiac implantable electronic device and the frequency of atrial fibrillation was the same as the general population. There was a higher frequency of MVP, and RBBB in FSHD when compared to the general population. Currently, there are no specific guidelines for cardiac screening in FSHD. In light of our findings and previously reported studies, it would be prudent to screen patients with FSHD for cardiac conduction dysfunction, regardless of age, degree of muscle weakness, or cardiac symptoms. Multi-center studies with serial ECG and echocardiographic long-term follow-up would be helpful to better ascertain risk of cardiac involvement in FSHD and to assess for progression of the cardiac abnormalities.

## Data Availability Statement

The raw data supporting the conclusions of this article will be made available by the authors, without undue reservation.

## Ethics Statement

The studies involving human participants were reviewed and approved by IRB. Written informed consent for participation was not required for this study in accordance with the national legislation and the institutional requirements.

## Author Contributions

AD-S: data curation, investigation, methodology, writing—original draft, and writing—review and editing. SN: data curation, investigation, and writing—review and editing. CAAC: visualization, investigation, writing—original draft, and writing—review and editing. KD-S and CGS: software and formal analysis. SR: data curation and investigation. NK, EKSL, TL, and PAB: writing—review and editing. VKS: conceptualization, validation, writing—review and editing, supervision, and funding acquisition. GL: validation, writing—review, and editing. MM: validation, writing—review and editing, supervision, project administration, and funding. All authors contributed to the article and approved the submitted version.

## Conflict of Interest

GL has received fees from the Pfizer Speaker's bureau. However, there was no involvement in this study in any way. VKS has served as a consultant to U-Health, GlaxoSmithKline, Price Waterhouse Coopers, Rhonda Gray, Dane Garvin, Philips, ResMed, Sorin Inc., and is working with Mayo Health Solutions and their industry partners on intellectual property related to sleep and cardiovascular disease. The Mayo Foundation has received a gift from the Philips Respironics Foundation for the study of sleep and cardiovascular disease. MM receives an honorarium to serve as associate editor of Neurology Genetics. However, none of these entities were involved in this study in any way. The remaining authors declare that the research was conducted in the absence of any commercial or financial relationships that could be construed as a potential conflict of interest.
